# Case of Hernia through the Thyroid Foramen

**Published:** 1833-07

**Authors:** 


THE LONDON

### Medical and Physical Journal.

413, VOL. LXX.] JULY 1833. [85, *New Series.*

"For many fortunate discoveries in medicine, and for the detection of numerous errors, the world is indebted to the rapid circulation of Monthly Journals; and there never existed any work to which the Faculty in Europe and America, were under deeper obligations than to the 'Medical and Physical Journal of London,' now forming a long but an invaluable series."—RUSH.

### ORIGINAL PAPERS.

### HERNIA THROUGH THE THYROID FORAMEN.

*Case of Hernia through the Thyroid Foramen.* Communicated by W. M., Esq., Surgeon.

*To the Editors of the London Medical and Physical Journal.*

GENTLEMEN: Should you be of my opinion, that, although those diseases which are the most common, are, in general, the most important, and demand more peculiarly the study of the medical practitioner, still that those disorders which are less frequent, although not absolutely rare, claim also no slight degree of attention, you will probably deem the following short report of a case of protrusion of the bowel through the foramen thyroideum, not wholly unworthy a place amongst the valuable records collected and preserved in the pages of the London Medical and Physical Journal.

It is not that this case presents any striking novelty, that I am induced to send you an extract of its chief points from my notes; but the conviction above expressed, that cases of seldom, though not absolutely rare occurrence, are profitably recorded; because, although ever on the alert to perceive and to balance the evidence of ordinary symptoms, occurring in ordinary cases, we are sometimes too apt to forget that nearly similar symptoms attend very dissimilar disorders; and, although well acquainted, theoretically and historically, with such cases, the probability of their occurrence in any individual instance being small, they are too often improperly left out of the calculation in practice.

A. B., a woman, sixty-five years of age, had been ailing for upwards of three years. She was habitually costive; indeed, so much so, that a week or ten days frequently passed without any evacuation from the bowels. She had been, from youth, accustomed to work much with her needle, and she attributed her indisposition to her protracted sedentary employment. Besides her dyspeptic complaints, she suffered considerably from pains in her head, and also from a numbness in her left leg, which had latterly increased. Within a few weeks of her death, she was attacked with violent spasms, which had formerly occurred only at intervals, but now they returned daily, commencing usually about nine o'clock in the evening, and lasting for several hours. It was probably during one of these spasmodic paroxysms that the gut made its escape.

On the 20th of February, I saw her for the first time, and attended her daily, with another surgeon, until her death, which took place on the 3d of March.

Costiveness being the most urgent symptom, aperients were administered by the mouth, and enemata frequently injected; but all to no purpose. The clysters speedily returned, unmixed with fæcal matter, and the stomach began to reject both food and medicines. The spasms were attempted to be relieved by the usual antispasmodic remedies, and the irritability of the stomach to be allayed by the application of a blister. A physician, who was consulted, was of opinion that a stone existed in the bladder, as she complained of great pain in the left groin, and of a frequent desire to make water. No stone, however, was felt by the sound; neither could anything unusual be detected in either inguinal region.

She died on Sunday morning, the 3d of March; and, permission being obtained from her friends, a post-mortem examination was made. The thoracic and abdominal viscera were healthy; the bladder somewhat contracted, and its parietes thicker than usual, but free from stone. On tracing the course of the intestines, it was, however, found that a small portion of the ileum had passed through the foramen thyroideum, and had formed very strong adhesions to the contiguous parts. The knuckle of intestine was so small, that it could not be expected to be discovered by external manual investigation; it had probably been long down, or at least occasionally protruded; but, during one of the violent spasmodic attacks to which the deceased was subject, a larger portion than usual descending through an aperture lessened by inflammation, it became strangulated, and caused her death.

The case was confessedly obscure, and yet, although the distinction might not have benefited the patient, I cannot but now think that materials existed for forming a more correct diagnosis, viz. the numbness of the left thigh and the pain in the groin, combined with long-continued costiveness and stercoraceous vomiting.

I remain, gentlemen, your obedient servant,

W. M., *Surgeon.*

[See some other cases of infrequent hernia, in No. 42, page 504, of this Journal.—EDITORS.]

